# Evidence, Theory and Context: Using intervention mapping to develop a worksite physical activity intervention

**DOI:** 10.1186/1471-2458-8-326

**Published:** 2008-09-22

**Authors:** Rosemary RC McEachan, Rebecca J Lawton, Cath Jackson, Mark Conner, Jennifer Lunt

**Affiliations:** 1 Institute of Psychological Sciences, University of Leeds, Leeds, UK; 2 School of Healthcare, University of Leeds, Leeds, UK; 3 Health and Safety Laboratory, Harpur Hill, Buxton, UK

## Abstract

**Background:**

The workplace is an ideal setting for health promotion. Helping employees to be more physically active can not only improve their physical and mental health, but can also have economic benefits such as reduced sickness absence. The current paper describes the development of a three month theory-based intervention that aims to increase levels of moderate intensity physical activity amongst employees in sedentary occupations.

**Methods:**

The intervention was developed using an intervention mapping protocol. The intervention was also informed by previous literature, qualitative focus groups, an expert steering group, and feedback from key contacts within a range of organisations.

**Results:**

The intervention was designed to target awareness (e.g. provision of information), motivation (e.g. goal setting, social support) and environment (e.g. management support) and to address behavioural (e.g. increasing moderate physical activity in work) and interpersonal outcomes (e.g. encourage colleagues to be more physically active). The intervention can be implemented by local facilitators without the requirement for a large investment of resources. A facilitator manual was developed which listed step by step instructions on how to implement each component along with a suggested timetable.

**Conclusion:**

Although time consuming, intervention mapping was found to be a useful tool for developing a theory based intervention. The length of this process has implications for the way in which funding bodies allow for the development of interventions as part of their funding policy. The intervention will be evaluated in a cluster randomised trial involving 1350 employees from 5 different organisations, results available September 2009.

## Background

Coronary heart disease and cancers are the biggest killers in developed countries [1,2]. Days lost to death, illness and informal care of people with coronary heart disease cost the UK economy £3 million, rising to £8 million when costs to the healthcare system are included [1]. Mortality rates for heart disease and cancer are falling, due to better and earlier treatment, but morbidity rates continue to rise as do risk factors such as obesity, diabetes and hypertension [1]. The causes of this increased morbidity are predominantly unhealthy lifestyles: smoking, unhealthy diets and physical inactivity. There is now convincing evidence that people who are physically active live longer and have lower morbidity [1]. Current epidemiological evidence links physical inactivity to heart disease [3-5], some cancers [6-8] and other chronic diseases, such as stroke [9,10]. Physical activity can also have positive effects on mental health [11]. Despite this evidence, the latest health survey for England [12] identified that only 37% of men and 25% of women engage in the recommended 30 minutes of moderate intensity activity, on five or more days of the week [13]. In the US and Canada around 48% of men and women engaged in the recommended levels of physical activity in 2005, which although more promising still suggests that over half the population are either inactive or not doing enough activity to benefit their health. [14,15]

UK Government strategy supports the promotion of health in the workplace [16]. This strategy goes beyond tackling work-related illness (e.g. musculoskeletal disorders and stress) and adopts a broader approach to workplace health in which workplaces are seen as sites for health promotion activities. Most adults spend half of their waking hours at work making the workplace an excellent setting for promoting health. Health promotion at work has demonstrated reduced sickness levels as well as economic benefits for organisations [17]. In addition to improving employee health, there is a strong business case for promoting physical activity. Many of the economic benefits are related to better health (e.g. reduced absenteeism) but less obvious benefits of physical activity have also been demonstrated, including reduced back pain, increased productivity, increased stress tolerance and improved decision-making [17].

The changing nature of work in the developed world, where manufacturing (e.g. steel production) and manual jobs (e.g. mining) are fewer and office based service industries dominate, means that large numbers of people are engaged in sedentary occupations. In these contexts the lack of physical activity can lead to work-related illness and prolonged recovery as well as increased morbidity and mortality. In this paper we describe the development of an intervention that targets employees in low physical activity occupations to encourage them to be more physically active. Government recommendations state that adults should aim to accrue at least 30 minutes of at least moderate intensity activity on at least five days of the week [13,18,19]. In light of the fact that employees have differing levels of baseline fitness the current project aimed at encouraging employees to *increase *the amount of physical activity they do on a daily basis with a view to working towards the recommended target for physical activity.

This intervention has been developed as part of a large cluster randomised control study in which more than 1350 employees from across 44 UK worksites (from a Local Council Authority, Teaching Hospital, Government Agency, Bus Company and University) were recruited between January and May 2008. The results of this trial will be available in late 2009.

An intervention mapping protocol [20] was used to ensure the intervention developed was grounded in theory. The utility of basing interventions upon sound theoretical frameworks is well expounded in the health promotion literature [e.g. [21,22]]. However, as yet the application of theory to the development of interventions has been less than optimal e.g. [23]. For example, a recent review of reviews of the effectiveness of interventions to prevent the spread of HIV found that none of the core review papers considered the role of theory in enhancing the effectiveness of the interventions [24]. In the development of interventions to change behaviour, theory serves three functions. First, knowledge of which theoretical constructs best explain or predict behaviour give a focus to the intervention, allowing the efforts to be concentrated on changing those psychological constructs central to behaviour change rather than those which are peripheral (the 'what' of the intervention). Second, theory can help to identify effective methods for changing these constructs and provide guidance as to 'how' behaviour may be changed. Finally, theory is important in allowing evaluation of 'why' change has occurred, whether for example, changes in behaviour are due to changes in one particular theoretical construct over another. This is important as it allows the elimination of elements of the intervention that focus on constructs which are less likely to lead to change therefore increasing the economy of the resulting intervention.

Intervention mapping is an iterative process encompassing 6 key stages: a needs assessment, the identification of outcomes and change objectives, the selection of theory based methods and practical strategies, the development of a programme plan, generation of adoption and implementation plan, and the generation of an evaluation plan. A detailed overview of how this protocol was used in the development of a worksite physical activity intervention is the focus of this paper.

## Methods

### Step one: Needs assessment, literature review, and focus groups

The first step of the intervention mapping process was to conduct a needs assessment and literature review. The evidence cited in the introduction serves as a justification for the focus on physical activity in the workplace and so the needs assessment is not discussed further. Pertinent literature was reviewed to identify a) which theoretical constructs best predict physical activity and b) which intervention strategies are found most frequently as part of successful interventions to increase physical activity. Finally focus groups were conducted with employees across three organisations to determine specific barriers and levers to activity in the workplace and to determine what would be feasible to implement in terms of a workplace intervention. Focus groups also informed the implementation process (step 5).

#### Participants and procedure

Sixty employees from the bus company, local authority and teaching hospital took part in 14 focus groups, including 21 bus drivers, 2 medical secretaries, 4 respiratory physiologists, 3 physiotherapists, 6 phlebotomists, 4 nurses, 3 home care managers, 7 teachers and teaching assistants and 9 staff from corporate services in the council. A discussion schedule was constructed which asked groups to think about the key barriers and levers to engaging in physical activity and to think about the most appropriate ways in which a physical activity intervention could be implemented in their worksite. Ethical approval was obtained from the University of Leeds Institute of Psychological Sciences Ethics committee and from the South Sheffield Local Research Ethics Committee. Written informed consent was obtained from all participants. The focus groups lasted between 30 and 90 minutes. Transcriptions of the focus groups were coded by RRCM.

### Step two: Identification of outcomes, performance objectives and change objectives

The next step involved specifying in detail the desired outcomes of the intervention. The overall desired outcome of the intervention was to increase physical activity. However, it was recognised that physical activity can occur in several different contexts (e.g. at home, at work) and have many different influences (e.g. personal, interpersonal and environmental [20]). Therefore specific intervention outcomes were specified for each context and each level of influence.

Second, performance objectives for each of the specified outcomes were specified. These are essentially a step by step checklist of what needs to happen in order to effect the outcomes. For example, if an outcome was to increase physical activity during work time, examples of performance objectives may be to 1: create intention to be physically active at work; and 2: identify appropriate opportunities to be active.

Finally, the objectives of the intervention need to be specified in terms of the actual change we need to see in the theoretical determinants of behaviour. This is important as it allows the intervention developer to identify exactly what needs to change in order to effect the performance objective, and ultimately the programme outcome. In order to achieve this, each performance objective is scrutinised separately. Intervention developers identify specific determinants which would be deemed useful in changing each performance objective. For example, if a performance objective was to create an intention to be physically active at work, appropriate theoretical determinants may be self-efficacy, attitudes and subjective norms [25,26]. The output of this process is a matrix of change objectives detailing what will be targeted in the intervention (see additional file 1 for an excerpt of the matrix for the current project, full matrices can be obtained from the authors on request). Although very time-consuming this process encourages intervention developers to be precise about which behaviours they should be targeting and what change objectives (actions) are required in order to achieve the performance objectives, and hence the outcomes.

### Step three: Selecting methods and practical strategies

After change matrices had been constructed the next stage was to select appropriate theoretical methods to change behaviour and translate these into practical strategies. For each determinant (e.g. self-efficacy) appropriate theoretical methods were identified from literature, and from guidance of Bartholemew et al [20] and an expert steering group. These were then translated into strategies suitable for implementation in the workplace. Decisions about what were suitable strategies were made in conjunction with the steering group, from feedback of key contacts within each organisation (e.g. Union officials, Management, Health and Safety Representatives) and from focus group data.

### Step four: Creating an organised programme plan

In the fourth step an organised programme plan was created. The expert steering group provided guidance as to scope of the intervention and the most appropriate channels for implementing the intervention. Up to this point a huge range of change objectives and potential strategies were identified, too many to target in one intervention. It was therefore necessary to filter all the strategies and change objectives into a number which were feasible to target in the current intervention. Key components of the intervention were selected based on feasibility of implementation and resource constraints. A timetable was created which specified which elements of the programme would be implemented when. Programme materials were developed and feedback from prospective worksites was elicited.

### Step five: Creation of an adoption and implementation plan

In step five a plan for how the intervention would be implemented was constructed. We decided to utilise employees from each of the participating worksites to act as local intervention 'facilitators'. These individuals were responsible for implementing the intervention. The use of local employees as facilitators was to ensure a local point of contact for those participating in the intervention, and to ensure that intervention could be tailored to the needs of the particular worksite. They also provided a source of informal peer leader influence [27]. In addition they meant that the intervention could be delivered in-house without the need for specialist input from outside organisations. Facilitators were enthusiastic and respected employees who were first identified via Management or Trade Union Officials to take on the role and were subsequently invited by the research team. Step five then, essentially involved repeating steps 2–3 of the intervention mapping process for behaviours we specifically wished to see from the facilitators. A key component of this stage was the production of a facilitator manual, development of facilitator training sessions and development of a protocol for keeping in touch with facilitators as they implemented the intervention.

### Step six: Creating an evaluation plan

In the final step of intervention mapping an evaluation plan was created. This is not in the scope of the current paper and so will be discussed elsewhere when reporting findings.

## Results

### Step one: Literature review

Literature was first reviewed to identify theoretical determinants useful in predicting and explaining physical activity. The Theory of Planned Behaviour was chosen as a particularly useful model upon which to base the intervention, having been very widely applied in the domain of exercise and physical activity e.g. [28,29]. Briefly, the Theory of Planned Behaviour (TPB) states that the proximal determinant of behaviour is intention, which encapsulates the motivational force that spurs an individual to action [25,30]. Intention in turn is held to be determined by attitudes toward the behaviour, subjective norms (perceptions of pressure to engage in the behaviour) and perceived behavioural control (the extent to which an individual believes they are capable of performing the behaviour). To the extent that perceived behavioural control reflects actual control over behaviour, then this factor can also directly predict behaviour. Reviews in this area have indicated that the TPB can typically account for between 42–45% of the variance in physical activity intentions and 27–36% of the variance of physical activity behaviour [28,31].

There is now increasing consensus that each of the constructs within the TPB may be better split into two components [32-34]. For example, attitude may be better conceptualised as including both affective (extent to which behaviour is seen as likeable and enjoyable) and instrumental (extent to which behaviour is seen as beneficial and useful) components. Subjective norms may be split into injunctive norms (what important others think) and descriptive norms (what important others do). Finally perceived behavioural control may be split into self-efficacy (an individual's confidence in their ability to perform the behaviour) and perceived control (control within the environment). Thus the current intervention contained strategies to change each of these subcomponent variables of the TPB.

The final determinant to be targeted in the current intervention was knowledge. This was added in recognition of the fact that a baseline level of knowledge about what physical activity is and how one might engage in physical activity was necessary if participants would be required to try to increase their own level of activity. To summarise, the key determinants which the current intervention targeted were: knowledge, affective and instrumental attitudes, subjective norms, self-efficacy and perceived control and intention.

Second the literature was reviewed to determine which types of strategies are most effective at changing physical activity behaviour. This was completed in November 2006. A large systematic review [35] and a review of reviews [36] of the effectiveness of interventions to increase physical activity were identified. Both reviews evaluated the effectiveness of interventions found within a range of settings (e.g. community and workplace) that were deemed relevant to the existing study.

In their systematic review, Kahn et al [35] identified a total of 95 interventions which they split into general categories according to the strategies used in each intervention. Not all of their intervention groupings were relevant to the current project (e.g. school based interventions), however, they found evidence to strongly recommend the use of the following types of interventions which may be of use in workplace settings.

1. Point of decision prompts. Having signs by lifts and escalators to encourage people to use the stairs.

2. Community wide campaigns, encompassing a wide range of intervention studies. Common elements of these types of studies were that they were 'multi-component' (e.g. containing many different activities), and included elements such as support and self-help groups, counselling, screening and education, community events and walking trails.

3. Social support in community settings. Again, a wide range of interventions were contained within this grouping. Common elements were the focus on building, strengthening and maintaining social networks through the use of strategies such as buddy systems, behavioural contracts, walking groups and discussion groups.

4. Individually adapted interventions. The authors found strong evidence to suggest that interventions tailored to an individual's specific interests where personal goals were set also appeared to be effective. Other common elements of these interventions included behavioural self-monitoring, building social support and reinforcement through self-reward.

5. Creation/enhanced access to places for physical activity with information and outreach. These interventions focused on environmental changes such as provision of gym/fitness equipment and walking trails. Other common elements in this theme included provision of screening, support or buddy programmes, seminar and counselling.

In order to ascertain more specific information about which types of intervention strategies showed the most success the 26 studies in the Kahn review that showed significant positive effects on physical activity were collated and examined further. Abraham and Michie [37] have developed a taxonomy of behaviour change techniques which have been used in interventions to change behaviour. This was used to code the specific strategies that made up the intervention described within each paper. The description of the intervention reported in each paper was read by the first author and coded using the taxonomy. The table of results recording the techniques used in each successful intervention can be found in additional file 2. Those techniques that were found most frequently in these successful interventions were 1) planning for social support/social change, 2) prompting intention formation, 3) providing instruction, 4) providing opportunities for social comparison, 5) prompting self-monitoring, and 6) prompting barrier identification. It is important to note that because most interventions in the review were multi-faceted interventions it is not possible to isolate those components within the intervention that have caused the change in behaviour. Thus it is not possible to conclude which of the components identified most frequently have the strongest influence on behaviour. For example, social support/change occurs very frequently but always alongside at least one other intervention component.

Further to this review we also drew on a review of reviews of public health physical activity interventions [36]. Although this review does include two reviews of workplace physical activity interventions [38,39] covering 49 quasi-experimental studies and experimental studies, the findings were inconclusive. The characteristics of studies showing an increase in physical activity at six months were:

• Health screening and counselling

• Follow-up and re-assessment of progress

• Encouragement to self-select moderate physical activities

• Opportunities to participate in supervised and unsupervised programmes of physical activities including aerobics, walking and cycling.

Although it is difficult to directly compare these elements with those identified above in the Kahn review, it seems there is a clear need to provide information and support, and opportunities for monitoring performance. Moreover, across the various settings and population groups in this review, there is convincing evidence that promoting moderate intensity activity such as walking and not requiring attendance at a facility are features of successful interventions. Based on these two reviews it was possible to identify important components for our intervention and to better understand the target behaviour of the intervention – the promotion of moderate intensity activities that are selected by the individuals themselves. Finally, it was clear from the literature review that there is, as yet, no strong evidence base for the design of workplace physical activity interventions.

### Step one: Focus group results

On completion of the literature review, the next step was to conduct focus groups amongst a selection of the participating organisations. Table 1 shows the most frequently cited barriers (left panel) and levers (right panel) to engaging in physical activity as identified in the focus group discussions. The figures to the left of each panel indicate the number of focus groups in which each of the barriers or levers was mentioned at least once. The most frequently cited barriers were a lack of time or competing demands on time. A lack of motivation (e.g. can't be bothered, I just want to relax when I'm not working) was another problem highlighted by many. For others, the barriers represented a lack of control (e.g. a lack of facilities) and many people referred to the negative consequences associated with exercising (e.g. being hot and sweaty, being embarrassed or the manager frowning on you taking breaks).

**Table 1:** Barrier and facilitators to engaging in physical activity identified in the focus groups

No. focus groups	Barrier	No. focus groups	Lever
12	I don't have any time	5	Doing things with other people
11	I'm too tired by the time I get home after work	4	Having access to gym at work
8	I just can't be bothered	4	Having a goal
8	There aren't any convenient facilities	4	Doing things you enjoy
7	It is more important for me to relax when I'm not working	3	Sign-posting (knowing where to go for info)
6	I have too many other commitments	3	Monitoring your activity levels
6	I don't like getting hot and sweaty	3	Planning
4	It's too dangerous to do things on my own	2	Monetary incentive
4	I'm too embarrassed	2	Having a routine
3	My manager and colleagues would frown on me taking a break	2	Being inspired by others

Participants referred to a different set of factors when discussing the factors that facilitate engagement in physical activity. Social support for physical activity was considered important (e.g. being inspired by and engaging in activity with other people). Doing things that were enjoyable was also perceived to support activity. Other factors focused on having a goal, planning and monitoring activity levels and making it part of a routine. Having access to local facilities was also mentioned frequently.

### Step two: identification of outcomes, performance objectives and change objectives

As mentioned above the overall target outcome of the current intervention was to increase physical activity. Currently it is recommended that adults should engage in moderate intensity physical activity for at least 30 minutes on at least 5 days of the week [13,18,19]. However, based on focus group discussions it was recognised that this may not be a realistic goal for many individuals who are very sedentary and may be regarded as somewhat unachievable given the 3 month timescale for the actual intervention. It was therefore decided that the objective of the intervention would be to 'increase levels of moderate intensity activity' with a view to achieving the recommended levels. An increase in moderate intensity activity was defined as any increase lasting at least 10 minutes, as this 10 minute period has been posited to be the minimum length required for health benefits [18]. Focusing on increasing levels of physical activity also meant that the intervention was inclusive to all employees within the worksite, particularly those sedentary employees for whom achieving the recommended levels of physical activity in a short period might be an unrealistic goal. Participants were encouraged to set graded goals for themselves and show cumulative increases in activity throughout the course of the intervention.

Due to differing work patterns and commitments within different organisations it was clear that it was not feasible for all employees to increase the amount of physical activity during their working day. For example, an office worker may be able to utilise lunch breaks or flexible working policies to engage in activity during the day, but a bus driver confined to their cab for 4 hours at a time with a limited break would not find this easy. Therefore the behavioural outcomes focused on increasing moderate intensity physical activity in three areas: a) *during work*, b) *in leisure time*, and c) *during commute to work*.

Interpersonal outcomes were also specified given the importance of social support interventions in achieving behaviour change identified in the Kahn review paper [35]. Interpersonal outcomes were to d) *encourage colleagues to engage in physical activity *and e) to *encourage friends and family to engage in physical activity*.

The next stage of the intervention mapping process was to specify the performance objectives for each of the programme outcomes. In a brainstorming session RL and RRCM listed all the steps that would need to be taken in order to achieve the programme outcomes. This process was informed by theoretical knowledge about determinants (e.g. intention) and facilitators of behaviour (e.g. goal setting, implementation intentions, [40]). This list was then validated by CJ and MC. The final list of performance objectives can be found in additional file 3.

Once the performance objectives had been specified the next stage was to cross these with the theoretical determinants to create matrices of 'change objectives'. For each performance objective we first identified which determinant might be appropriate to achieve it. Then we specified what change we would need in the determinant in order to effect the performance objective. For example, to allow individuals to monitor current levels of activity (performance objective 6), it was deemed that intention, self-efficacy and knowledge should be targeted. Table 2 contains selected examples of change objectives for four different performance objectives.

**Table 2:** Examples of change objectives for selected performance objectives

Performance objective	Determinant	Change objective(s)
P.O. 6. Monitor current levels of activity in work-time/leisure time	Intention	Increase intention to monitor current activity levels
	Self-efficacy	Express confidence in monitoring current and ongoing activity levels at work Demonstrate ability to monitor current and ongoing activity levels at work
	Knowledge	Know what counts as 'activity' Know how to complete monitoring form

P.O.8 Manage competing demands in work	Attitude	Increase recognition of importance of physical activity
	Self-efficacy	Express confidence in managing competing demands at work Demonstrate ability to manage competing demands at work
	Subjective norms	Manage others expectations of you at work
	Knowledge	Know demands on your time

P.O20. Overcome barriers to increasing physical activity in commute to work	Self-efficacy	Get confidence in ability to overcome barriers
	Subjective norms	Enlist others to help overcome barriers
	Knowledge	Identify barriers and ways to overcome them

PO36. Audit family and friends' preferences for physical activity	intention	Intend to find out what types of physical activity family want to engage in
	Self-efficacy	Have the ability to convince others about importance of doing physical activity
	Subjective norms	Enlist support from family and friends
	Knowledge	Know what other people who are close to you want to engage in

### Step three: identifying theoretical methods and practical strategies

The third stage of the intervention mapping process involved identifying appropriate theoretical methods which are thought to change theoretical determinants. Bartholomew and colleagues [20] have summarised which types of theoretical methods are most appropriate for different theoretical determinants (chapter 7). This was used as a guide in the current process.

The intervention strategies identified as being associated with success in the literature review were also kept in mind when strategies for the current intervention were being developed (for example, self-monitoring, barrier identification). However, care was taken to ensure that all strategies included were based on sound theoretical methods. In addition, the results of the focus groups were reviewed to ensure that the practical strategies identified to implement the theoretically changed methods were appropriate and acceptable to the target group. For example, one strategy identified was having group discussions in which barriers to physical activity were discussed. However, it became clear from focus group discussions that it was not possible to get groups of employees together in the different worksites and that this component would fail as a result. Finally, the proposed strategies were presented to key stakeholders from the different types of organisations. Examples of theoretical methods and strategies related to self-efficacy change objectives for performance objective 6 (monitoring current levels of activity) can be found in Table 3. For example, one change objective was for individuals to express confidence in monitoring current and ongoing activity levels at work. Theoretical methods deemed useful here were guided practice (explaining how to go about monitoring activity), enactment (actually making the individual go through this process) and persuasive communications. In light of these theoretical methods we decided one strategy would be to provide participants with a 'work-book' where they could record how much activity they performed on different days of the weeks and at different times. It was decided that this might be appropriate in a leaflet format.

**Table 3:** Examples of strategies for self-efficacy change objectives for performance objective 6 (monitoring currently levels of physical activity in work)

Change objective	Theoretical method(s)	Strategy
Express confidence in monitoring current and ongoing physical activity levels at work	Guided practice Enactment Persuasive communications	Workbook and record of current activity. Flowchart that takes people in different directions according to their physical activity levels. This could be via a leaflet or website, but must require active responses from participant.

Express confidence in managing competing demands at work Demonstrate ability to manage competing demands at work	Persuasive communications Enactment Modelling	Write down barriers/competing demands and ways of overcoming them. Have role model stories of people successfully managing competing demands

### Stage four: creating an organised programme plan

The first step in stage four was to decide the scope and limits of the current intervention. Hillsdon et al. [36] comment that many physical activity interventions are delivered at the level of the individual and such interventions may not be economically viable or efficacious for achieving changes across a large population. An expert steering group consisting of health and work psychologists, occupational health specialists, and a physician decided on the core principles of the intervention. Contacts within the participating organisations were also consulted. They had three requirements. The intervention should be 1) flexible enough to be delivered across different sizes and types of organisation, 2) sustainable without the direct input of an expert group, 3) problem focused (i.e. able to work within the restraints of different work patterns and environments). The focus group results helped us to understand the different working contexts and cultures of each organisation and to identify appropriate intervention components. Finally, in order to map onto the three characteristics of successful interventions identified by Kahn et al [26] an informational, behavioural/motivation and environmental component to the intervention was deemed necessary. Thus, the name 'AME for Activity' (Awareness, Motivation and Environment) was coined.

Next it was necessary to translate the strategies into organised programme components or methods for delivering the strategies. The defining characteristic of the intervention was that it would be delivered 'in-house' by nominated local facilitators and would take no more than five hours each month (for a three month period). The delivery of materials and intervention components via a local facilitator was entirely pragmatic and based on the requirement that this intervention could be delivered in a variety of workplaces without expert input. This also ensured the sustainability of the intervention beyond the involvement of the research team. In addition, as the intervention was to be delivered over a three month period, a drip-feed approach was used in which different messages or activities would be the focus in different weeks. This was to ensure that employees did not habituate to, or cease to register, any of the intervention materials. The theme for the first month focused on health benefits of physical activity, the second month focused on mental health benefits and the final month focused on benefits of physical activity for leading a happy and fulfilling life. These themes were selected to reflect the different types of benefits that physical activity can have on health and life and acknowledging the influence of affective processes on behaviour [e.g. [41,42]].

Due to the large number of change objectives and resulting strategies it was necessary to refine strategies into a manageable number of key modes of delivery which could be implemented by the local facilitators. Key considerations in the selection of delivery modes were budget, whether the components could be delivered in all the different worksites, and the amount of time/skill needed by each facilitator to implement them. Four members of the research team (RRCM, RJL, CJ and MC) each reviewed the list of change objectives and strategies and identified the key components that identified the majority of the strategies. Ten components were identified which, although not mutually exclusive, fitted into three themes of 'awareness', 'motivation' and 'environment', (see Table 4).

**Table 4:** Key components of AME for ACTIVITY intervention

Awareness	Motivation	Environment
Launch week	Setting personal targets	Management support
Interactive leaflets*	Making plans	Newsletters
Posters	Self-monitoring	Reminders
Knowledge quiz	Team challenges	

*Leaflets containing tasks and requiring active processing

The intervention components differed according to whether they were delivered on an individual level (e.g. leaflets, quiz, plans and targets, self monitoring, newsletters, reminders) or whether they were delivered at a group level (e.g. team challenges). The remaining components drew on existing communication strategies within our collaborating organisations (e.g. posters, management support letters). Emphasis was placed on engaging with group activities and team challenges on offer within the workplace, but if employees did not choose to do this then at the minimum they would receive materials which could be completed on their own. These materials included 3 leaflets (each distributed four weeks apart and covering a different theme) and a monitoring tool which allowed them to keep track of how much activity they achieved each month. These, along with the posters were designed by a professional graphic design agency. A logo was designed to provide a unifying corporate theme to the remainder of the materials which were developed by the research team.

During the process of developing and designing each of these components of the intervention the value of the intervention mapping process was realised. The content of these materials was driven by strategies previously identified. For example, role model stories were integrated into leaflets and newsletters. Participants were encouraged in each leaflet to identify barriers to physical activity and ways of overcoming them.

The final intervention consisted of these 10 components designed to be delivered in a systematic fashion over a 12 week period. The intervention pack for each worksite contained copies of three different interactive leaflets (which encouraged participants to set targets, make plans and provided feedback on their progress) and a 'keeping track' monitoring tool (in the form of an A5 magnet with erasable pen for recording activity levels). These materials were distributed to all participating employees. In addition the intervention pack contained copies of 8 different A3 coloured posters, and electronic templates for newsletters, reminders, letters of management support, quizzes and instructions on how to run team challenges. The suggested timetable for the delivery of each of these intervention components can be found in additional file 4.

### Stage five: creation of an adoption and implementation plan

Once the intervention strategies had been finalised and their feasibility assessed the next stage was to create a plan for the adoption and implementation of the intervention amongst the target group. A crucial element of this was to ensure that facilitators received the correct training and instruction in order to implement the intervention. Thus, steps 2 and 3 of the intervention mapping process were repeated to focus on the behaviours required from the facilitator to implement the intervention. The outcome of this was a comprehensive facilitator manual including step by step instructions about how to implement the intervention and a suggested timetable for implementing each of the key components. An implementation plan was also developed to provide facilitators with ongoing support throughout the implementation of the intervention. This is currently underway. The research team first contacts the facilitator to arrange a convenient time for the intervention to launch. Then a second member of the research team (CJ) contacts facilitators at monthly intervals to assess progress. As part of the process facilitators are asked to complete an 'intervention' log detailing which components of the intervention are delivered each week. This log will be used as part of a process evaluation on conclusion of the evaluation to determine which elements of the intervention are consistently implemented and which are not.

## Discussion

The process of developing this intervention began before the funding for the research was agreed. However, at this early stage only a commitment to covering areas of awareness, motivation and environment was specified. The process of developing the intervention using intervention mapping is described in this paper. This work was time consuming and challenging, involving literature reviewing, focus group work and team meetings, and taking a year to complete. In fact, we estimate that this process involved 8 months full-time work of the Research Fellow and 6 weeks full-time work by the Research Team. The AME for Activity intervention produced as a result of this work is theoretically grounded and evidence based. The addition of focus groups and consultation with managers and Union officials to the development process has also helped to produce an intervention that is pragmatic and feasible to deliver within a variety of different workplace settings. The evaluation of the success of the intervention will take place during a large cluster-randomised control trial and will be reported in 2009, at which point we hope to have objective measures of health, measures of well-being and self-report measures of physical activity, from a sample of 900 employees taken at two points in time, 12 months apart.

Although we found intervention mapping to be useful, a limitation is the time-consuming nature of the protocol. Our experience in this regard was similar to other researchers who have used the intervention mapping protocol e.g. [43-45]. The creation of the matrices of change objectives for some 57 performance objectives was particularly time consuming and resulted in an overwhelming amount of information about what should be targeted in the intervention. We were not able to address all these change objectives in the current intervention, and had to spend time filtering the change objectives and strategies to those which could be pragmatically achieved. Kwak et al., comment that the intervention mapping protocol is typically applied to simple and uni-dimensional behaviours and can become unwieldy when applied to multi-dimensional behaviours such as those involved in weight gain prevention and physical activity [43]. In the development of their workplace intervention to prevent weight gain amongst young adults they decided not to create matrices of change objectives, instead focusing the specification of their performance objectives on clear theoretical steps which they then identified strategies to change. This may be a useful short-cut for researchers developing interventions targeting complex and multi-dimensional behaviours. However, despite the time-consuming nature of the protocol we believe it allowed us to create a comprehensive intervention package that was tightly focused and theory based.

It is important to consider the implications of this extensive intervention mapping process for the way intervention research is funded. This three year project specified an intervention development period of one year. However, the funding of randomised and cluster-randomised control trials is often based on the premise that the intervention is well specified at the proposal stage. In the public health arena it is difficult to attract funding to develop an intervention. This means that 1) the development work is carried out without the necessary funding and/or 2) the intervention is not given sufficient consideration, is not theoretically grounded or does not meet the needs of the end user (e.g. the target population). This may help to explain why interventions are often not theoretically grounded or evidence based [23]. This lack of funding also means that although intervention mapping may represent the ideal for intervention development it may not always be feasible and we would not recommend embarking on the process without the necessary time and resources. Moreover, as health psychologists, the terminology employed in the guidance material for intervention mapping was familiar. However, without this expertise it is likely that we would have found the intervention mapping process more difficult. Thus, careful consideration should be given to deciding the membership of the intervention development team.

The intervention is currently being delivered across 22 worksites in the UK (with a further 22 worksites acting as controls). Due to the time constraints of the project we were unable to perform a full pilot of the intervention (i.e. including the full 12 month follow up). However, we did follow Medical Research Council guidelines for the implementation of complex interventions [46] as closely as possible and trialled the intervention in three 'test' worksites in November – December 2007 before rolling out the intervention to the remaining worksites in February – May 2008. All three facilitators from these worksites were interviewed by phone and the feasibility of the intervention discussed with them. Feedback was positive and issues that were raised in these interviews were helpful in formulating the one-day facilitator training for the main trial that took place in December 2007. For example, facilitators expressed some unease about how to work with people in their group who were resistant to change. In response to this, we ran a session during the training day on overcoming barriers to change. We also reassured facilitators that it was not part of their remit to 'force' change. We invited facilitators within the test worksites to attend the training day for the remaining facilitators in order to give their experiences of implementing the intervention. This was found to be an extremely useful way of imparting knowledge to facilitators and building enthusiasm for the intervention. Before the start of the main trial we were able to gain feedback on all three months of the intervention. Feedback from these 'test' worksites at this stage indicated no changes were required to any of the materials or the facilitators' manual.

It is important to acknowledge the limitations of this intervention. Although encompassing some minor changes to the environment (e.g. having a local facilitator and fostering an environment supportive of physical activity), this intervention did not include changes to the physical environment (e.g. shower facilities or walking routes) or to systems of work (e.g. physical activity breaks). These require some considerable commitment and resources from management and without evidence of the economic benefits of physical activity interventions our collaborators indicated that they were not willing to make such investments. In the future consideration should be given to tackling these environmental changes within such intervention programmes.

## Conclusion

The current paper describes the development and key components of the 'AME for Activity' intervention using intervention mapping. Although a long process, the intervention mapping protocol allowed us to develop a theory based, low resource intervention which can be delivered in the workplace by local employees.

## Competing interests

The authors declare that they have no competing interests.

## Authors' contributions

RRCM and RJL completed the intervention mapping process, conducted the literature review and drafted the manuscript. RRCM conducted the focus groups. CJ and MC participated in the intervention mapping process and helped to draft the manuscript. JL provided expert guidance in the early stages of intervention development and provided comments on the manuscript. All authors read and approved the manuscript.

## Pre-publication history

The pre-publication history for this paper can be accessed here:



## Supplementary Material

Additional file 1 **Excerpt from change matrices (word document).** Excerpt from matrix of change objective for behavioural outcome 1. Increasing physical activity at work. Tabular data. Click here for file

Additional file 2 **Coding successful interventions (word document).** Content of successful interventions identified in Kahn et al (2001) using Abraham & Michie (in press) coding frame. Tabular data and reference list. Click here for file

Additional file 3 **Programme outcomes and Performance objectives (word document).** Programme outcomes and performance objectives. Tabular data. Click here for file

Additional file 4 **Suggested timetable (word document).** Suggested timetable for intervention components. Tabular data. Click here for file
