# Supplementary material for: Evidence, Theory and Context: Using intervention mapping to develop a worksite physical activity intervention
Source: BMC Public Health. 2008 Sep 22;8:326. doi: 10.1186/1471-2458-8-326 (PMC2567979; doi:10.1186/1471-2458-8-326)
Supplement: Additional file 1 — Excerpt from change matrices (word document). Excerpt from matrix of change objective for behavioural outcome 1. Increasing physical activity at work. Tabular data. [file 1471-2458-8-326-S1.doc]

Additional file 1. Excerpt from matrix of change objective for behavioural outcome 1. Increasing physical activity at work.

| Performance objective | Intention | Self-efficacy | Affective and instrumental attitudes | Social Norms | Knowledge |
| --- | --- | --- | --- | --- | --- |
| 1. Create realistic goal |  |  | ATT1. Believe that there will be benefits and positive feelings associated with engaging in physical activity at work. |  | K1. Describe the benefits of physical activity for health, wellbeing and performance  K2. Describe how much activity to engage in for health, wellbeing and performance  K3. Describe the risks associated with not engaging in physical activity  K4. Know what realistic goal is  K5 Know what types of physical activities which can be performed at work will benefit health |
| 1. Create intention |  | SE1. Express confidence in achieving realistic goal of PA at work | as ATT 1 | SN1. Recognise that others want you to engage in physical activity at work.  SN2. Recognise that other employees engage in physical activity at work.  SN3. Identify yourself with a realistic ‘prototype’ who engages in physical activity at work. |  |
| 1. Link with other valued goals |  | SE2. Express confidence in linking physical activity at work with other activities  SE3. Demonstrate performance of enjoyable activities at work | ATT2. Believe that physical activity be effective in helping to achieve other desired goals in life. |  | K6. Know what other goals being physically active at work can influence |
| 1. Identify possibilities and opportunities | I1. Increase motivation to identify possibilities and opportunities to engage in physical activity at work | SE4a) Express confidence in identifying appropriate opportunities and activities for physical activity in work  SE4b) Demonstrate ability to identify appropriate opportunities and activities for physical activity in work. |  |  | K7. Know situations in which can perform physical activity in work  K8. Can identify times to engage in physical activity at work. |
| 1. Select appropriate activities |  | SE5. Express confidence in performing physical activity at work  SE6a. Express confidence in seeking advice about physical activity at work  SE6b. Demonstrate ability to seek advice about physical activity in work. | ATT3. Think that different physical activities are enjoyable and beneficial | SN4. Select physical activities that others want to do in work | K9. Be aware of own limits  K10. Know how to perform selected activities |
| 1. Monitor current levels of activity | I2. Increase intention to monitor current levels of physical activity at work | SE7a. Express confidence in monitoring current and ongoing activity levels at work.  SE7b. Demonstrate ability to monitor current and ongoing physical activity levels at work. |  |  | K11. Know what physical activity is  K12. Know how to complete monitoring form  K13. Know what scores mean for health |
| 1. Set personal targets | I3. Want to set targets above current physical activity levels at work | SE8. Demonstrate ability to set achievable and appropriate targets for physical activity at work  SE9. Express confidence in achieving targets for physical activity at work. |  |  |  |
| 1. Manage competing demands |  | SE10a. Express confidence in managing competing demands for physical activity at work.  SE10b. Demonstrate ability to manage competing demands for physical activity at work. | ATT4. Believe that engaging in physical activity at work is important | SN5. Manage others expectations of you at work | K14. Know demands on your time at work |
| 1. Overcome barriers |  | SE11a. Express confidence in overcoming practical barriers of physical activity at work.  SE11b. Demonstrate ability to overcome practical barriers of physical activity at work. | ATT5. Believe that barriers to engaging in physical activity at work are not in surmountable |  | K15. Know what the major barriers to physical activity at work are |
| 1. Create implementation intentions | I4. Be motivated to perform activity and willing to create implementation intentions for physical activity at work | SE12. Demonstrate ability to set appropriate plans for physical activity at work. | ATT6. Believe that performing specific plan will help achieve goal of performing physical activity in work |  | K16. Know how to specify implementation intentions |
| 1. Maintain progress |  |  | ATT7. Focus on positive experience of engaging in physical activity in work time |  |  |
| 1. Provide feedback on progress | I5. Be motivated to receive feedback about physical activity levels at work | SE13a. Express confidence in providing feedback to self about levels of physical activity at work.  SE13b. Demonstrate ability to provide self with feedback at work. |  | SN6. Compare your progress with others | K17. know how to give feedback  K18. Know what changes mean for health |
| 1. Continue monitoring and revise goals | I6 Intend to set new targets for physical activity in work when existing ones achieved. | SE14a. Express confidence in recognising achievements for physical activity at work.  SE14b. Demonstrate ability to reward achievements for physical activity at work.  SE15. Express confidence in coping with disappointment  SE16. Demonstrate ability to revise goals (up or down)  SE17a. Express confidence in revising planning and overcoming barriers  SE17b. Demonstrate ability to revise planning and overcoming barriers. |  |  |  |
