# Supplementary material for: Evidence, Theory and Context: Using intervention mapping to develop a worksite physical activity intervention
Source: BMC Public Health. 2008 Sep 22;8:326. doi: 10.1186/1471-2458-8-326 (PMC2567979; doi:10.1186/1471-2458-8-326)
Supplement: Additional file 2 — Coding successful interventions (word document). Content of successful interventions identified in Kahn et al (2001) using Abraham & Michie (in press) coding frame. Tabular data and reference list. [file 1471-2458-8-326-S2.doc]

Additional File 2. Content of successful interventions identified in Kahn et al (2001) using Abraham & Michie (in press) coding frame

|  | Study Number | | | | | | | | | | | | | | | | | | | | | | | | | | |
| --- | --- | --- | --- | --- | --- | --- | --- | --- | --- | --- | --- | --- | --- | --- | --- | --- | --- | --- | --- | --- | --- | --- | --- | --- | --- | --- | --- |
|  | Total | [1] | [2] | [3] | [4] | [5] | [6] | [7] | [8] | [9] | [10] | [11] | [12] | [13] | [14] | [15] | [16] | [17] | [18] | [19] | [20] | [21] | [22] | [23] | [24] | [25] | [26] |
| 1 Provide general information | 10 |  |  | x |  |  |  | x | x |  | x |  |  | x | x |  |  |  | x | x |  |  |  |  |  | x | x |
| 2 Provide information on consequences | 5 |  |  |  |  | x | x |  |  |  |  |  |  |  | x |  |  |  |  |  |  |  |  |  |  | x | x |
| 3 Provide information about others’ approval | 0 |  |  |  |  |  |  |  |  |  |  |  |  |  |  |  |  |  |  |  |  |  |  |  |  |  |  |
| 4 Prompt intention formation | 16 |  | x | x | x | x | x |  | x | x | x | x |  | x |  | x | x | x | x |  |  |  |  |  | x |  | x |
| 5 Prompt specific goal setting | 4 |  |  |  |  |  |  |  |  | x |  |  |  |  |  | x |  |  |  |  |  | x |  |  | x |  |  |
| 6 Set graded tasks | 11 |  |  |  | x | x | x |  | x | x | x | x |  |  | x |  | x |  | x |  |  |  |  | x |  |  |  |
| 7 Prompt barrier identification | 13 |  |  | x | x | x | x |  | x | x | x |  |  |  |  |  | x |  | x | x |  | x | x |  |  |  | x |
| 8 Agree behavioural contract | 4 |  |  |  | x |  |  |  |  | x |  |  |  | x |  |  |  |  |  | x |  |  |  |  |  |  |  |
| 9 Prompt review of behavioural goals | 0 |  |  |  |  |  |  |  |  |  |  |  |  |  |  |  |  |  |  |  |  |  |  |  |  |  |  |
| 10 Provide Instruction | 16 |  |  | x | x |  | x | x | x | x |  | x |  | x | x | x | x |  |  |  | x | x | x |  | x | x |  |
| 11 Model/ demonstrate the behaviour | 5 |  |  |  |  |  |  |  | x | x |  |  |  |  | x |  |  |  |  | x |  |  |  |  |  | x |  |
| 12 Prompt practice | 9 | x |  |  | x |  |  |  | x |  |  |  |  | x | x |  |  |  | x | x |  |  |  |  |  | x | x |
| 13 Prompt self-monitoring of behaviour | 14 |  |  |  | x |  | x |  | x | x | x | x |  |  | x | x | x |  |  | x |  |  | x | x | x |  | x |
| 14 Provide feedback on performance | 10 |  |  | x | x |  | x | x |  |  |  | x |  | x | x |  |  |  |  | x |  |  | x |  | x |  |  |
| 15 Provide general encouragement | 9 |  | x |  |  | x |  | x |  |  |  |  | x | x |  |  |  |  |  | x |  |  |  | x | x |  | x |
| 16 Provide contingent rewards | 9 |  |  |  | x |  | x |  | x | x |  |  |  |  |  | x | x |  | x |  |  |  |  | x |  | x |  |
| 17 Teach to use prompts/ cues | 1 |  |  |  |  |  |  |  |  |  |  |  |  |  |  |  | x |  |  |  |  |  |  |  |  |  |  |
| 18 Use follow up prompts | 4 |  |  |  |  |  |  |  |  |  |  | x |  | x |  | x |  |  |  |  | x |  |  |  |  |  |  |
| 19 Provide opportunities for social comparison | 15 | x |  |  | x | x | x |  | x |  | x | x |  | x | x |  |  |  | x | x |  | x |  | x |  | x | x |
| 20 Plan social support/ social change | 18 | x |  | x | x | x |  |  | x | x |  |  |  | x | x | x |  |  | x | x | x | x | x | x | x | x | x |
| 21 Prompt identification as role model | 0 |  |  |  |  |  |  |  |  |  |  |  |  |  |  |  |  |  |  |  |  |  |  |  |  |  |  |
| 22 Prompt self talk | 2 |  |  | x |  |  |  |  |  |  |  |  |  |  |  |  | x |  |  |  |  |  |  |  |  |  |  |
| 23 Relapse prevention | 4 |  |  | x |  |  |  |  | x |  |  |  |  |  |  |  |  | x | x |  |  |  |  |  |  |  |  |
| 24 Stress management | 2 |  |  |  |  |  | x |  |  |  |  |  |  | x |  |  |  |  |  |  |  |  |  |  |  |  |  |
| 25 Motivational interviewing | 0 |  |  |  |  |  |  |  |  |  |  |  |  |  |  |  |  |  |  |  |  |  |  |  |  |  |  |
| 26 Time management | 0 |  |  |  |  |  |  |  |  |  |  |  |  |  |  |  |  |  |  |  |  |  |  |  |  |  |  |

Notes:

Column 2 (Total) sums the number of times each technique was identified across the successful interventions.

Reference List

1. Blair SN, Piserchia PV, Wilbur CS, Crowder JH: **A Public-Health Intervention Model for Work-Site Health Promotion - Impact on Exercise and Physical-Fitness in A Health Promotion Plan After 24 Months.** *Jama-Journal of the American Medical Association* 1986, **255:**921-926.

2. Cardinal BJ, Sachs ML: **Prospective Analysis of Stage-Of-Exercise Movement Following Mail-Delivered, Self-Instructional Exercise Packets.** *American Journal of Health Promotion* 1995, **9:**430-432.

3. Chen AH, Sallis JF, Castro CM, Lee RE, Hickmann SA, Williams C, Martin JE: **A home based behavioral intervention to promote walking in sedentary ethnic minority Women: Project WALK.** *Women's Health: Research on Gender, Behavior and Policy* 1998, **4:**19-39.

4. Coleman KJ, Raynor HR, Mueller DM, Cerny FJ, Dorn JM, Epstein LH: **Providing sedentary adults with choices for meeting their walking goals.** *Preventive Medicine* 1999, **28:**510-519.

5. Dunn AL, Marcus BH, Kampert JB, Garcia ME, Kohl HW, Blair SN: **Comparison of lifestyle and structured interventions to increase physical activity and cardiorespiratory fitness - A randomized trial.** *Jama-Journal of the American Medical Association* 1999, **281:**327-334.

6. Foreyt JP, Goodrick GK, Reeves RS, Raynaud AS, Darnell L, Brown AH, Gotto AM: **Response of Free-Living Adults to Behavioral Treatment of Obesity - Attrition and Compliance to Exercise.** *Behavior Therapy* 1993, **24:**659-669.

7. Jarvis KL, Friedman RH, Heeren T, Cullinane PM: **Older women and physical activity: Using the telephone to walk.** *Womens Health Issues* 1997, **7:**24-29.

8. Jeffery RW, Wing RR, Thorson C, Burton LR: **Use of personal trainers and financial incentives to increase exercise in a behavioral weight-loss program.** *Journal of Consulting and Clinical Psychology* 1998, **66:**777-783.

9. Jette AM, Lachman M, Giorgetti MM, Assmann SF, Harris BA, Levenson C, Wernick M, Krebs D: **Exercise - It's never too late: The Strong-for-Life program.** *American Journal of Public Health* 1999, **89:**66-72.

10. Kanders BS, Ullmannjoy P, Foreyt JP, Heymsfield SB, Heber D, Elashoff RM, Ashley JM, Reeves RS, Blackburn GL: **The Black-American Life-Style Intervention (Bali) - the Design of A Weight-Loss Program for Working-Class African-American Women.** *Journal of the American Dietetic Association* 1994, **94:**310-312.

11. King AC, Haskell WL, Taylor CB, Kraemer HC, Debusk RF: **Group-Based Vs Home-Based Exercise Training in Healthy Older Men and Women - A Community-Based Clinical-Trial.** *Jama-Journal of the American Medical Association* 1991, **266:**1535-1542.

12. Marcus BH, Emmons KM, Simkin-Silverman LR, Linnan LA, Taylor ER, Bock BC, Roberts MB, Rossi JS, Abrams DB: **Evaluation of motivationally tailored vs. standard self-help physical activity interventions at the workplace.** *American Journal of Health Promotion* 1998, **12:**246-253.

13. Mayer JA, Jermanovich A, Wright BL, Elder JP, Drew JA, Williams SJ: **Changes in Health Behaviors of Older Adults - the San-Diego-Medicare-Preventive-Health-Project.** *Preventive Medicine* 1994, **23:**127-133.

14. Mcauley E, Courneya KS, Rudolph DL, Lox CL: **Enhancing Exercise Adherence in Middle-Aged Males and Females.** *Preventive Medicine* 1994, **23:**498-506.

15. Noland MP: **The Effects of Self-Monitoring and Reinforcement on Exercise Adherence.** *Research Quarterly for Exercise and Sport* 1989, **60:**216-224.

16. Owen N, Lee C, Naccarella L, Haag K: **Exercise by Mail - A Mediated Behavior-Change Program for Aerobic Exercise.** *Journal of Sport Psychology* 1987, **9:**346-357.

17. Peterson TR, Aldana SG: **Improving exercise behavior: An application of the stages of change model in a worksite setting.** *American Journal of Health Promotion* 1999, **13:**229-232.

18. Wing RR, Jeffery RW, Pronk N, Hellerstedt WL: **Effects of a personal trainer and financial incentives on exercise adherence in overweight women in a behavioral weight loss program.** *Obesity Research* 1996, **4:**457-462.

19. Avila P, Hovell MF: **Physical-Activity Training for Weight-Loss in Latinas - A Controlled Trial.** *International Journal of Obesity* 1994, **18:**476-482.

20. Jason LA, Greiner BJ, Naylor K, Johnson SP, van Egeren L: **A large-scale, short-term, media-based weight loss program.** *American Journal of Health Promotion* 1991, **5:**432-437.

21. King AC, Frederiksen LW: **Low-Cost Strategies for Increasing Exercise Behavior - Relapse Preparation Training and Social Support.** *Behavior Modification* 1984, **8:**3-21.

22. King AC, Taylor CB, Haskell WL, Debusk RF: **Strategies for Increasing Early Adherence to and Long-Term Maintenance of Home-Based Exercise Training in Healthy Middle-Aged Men and Women.** *American Journal of Cardiology* 1988, **61:**628-632.

23. Kriska AM, Bayles C, Cauley JA, Laporte RE, Sandler RB, Pambianco G: **A Randomized Exercise Trial in Older Women - Increased Activity Over 2 Years and the Factors Associated with Compliance.** *Medicine and Science in Sports and Exercise* 1986, **18:**557-562.

24. Lombard DN, Lombard TN, Winett RA: **Walking to Meet Health Guidelines - the Effect of Prompting Frequency and Prompt Structure.** *Health Psychology* 1995, **14:**164-170.

25. Simmons D, Fleming C, Voyle J, Fou F, Feo S, Gatland B: **A pilot urban church-based programme to reduce risk factors for diabetes among Western Samoans in New Zealand.** *Diabetic Medicine* 1998, **15:**136-142.

26. Wankel LM, Yardley JK, Graham J: **The effects of motivational interventions upon the exercise adherence of high and low self-motivated adults.** *Canadian Journal of Applied Sport Sciences / Journal Canadien des sciences appliquees au sport* 1985, **10:**147-156.
