# Supplementary material for: Evidence, Theory and Context: Using intervention mapping to develop a worksite physical activity intervention
Source: BMC Public Health. 2008 Sep 22;8:326. doi: 10.1186/1471-2458-8-326 (PMC2567979; doi:10.1186/1471-2458-8-326)
Supplement: Additional file 3 — Programme outcomes and Performance objectives (word document). Programme outcomes and performance objectives. Tabular data. [file 1471-2458-8-326-S3.doc]

## Additional file 3. Programme outcomes and performance objectives

| Programme outcome | Performance objectives |
| --- | --- |
| a. Increase performance of moderate intensity physical activity in work  b. Increase performance of moderate intensity physical activity in leisure time | 1. Create realistic goal 2. Create intention 3. Link with other valued goals 4. Identify possibilities and opportunities 5. Select appropriate activities 6. Monitor current levels of activity 7. Set personal targets 8. Manage competing demands 9. Overcome barriers 10. Create implementation intentions 11. Maintain progress 12. Provide feedback on progress 13. Continue monitoring and revise goals |
| c. Increase performance of moderate intensity physical activity in commute to work | 1. Create realistic goal 2. Audit current travel plans 3. Identify possibilities and opportunities 4. Create intention 5. Link with other valued goals 6. Develop specific plans 7. Overcome barriers 8. Implement plans 9. Provide feedback 10. Revisit (breaking habit) |
| d. encourage colleagues to engage in physical activity | 1. Generate awareness 2. Generate interest 3. Identify appropriate opportunities 4. Plan time for discussion 5. Be supportive 6. Be a role model 7. Suggest ways of overcoming barriers 8. Set team goals/ targets 9. Provide visible feedback 10. Encourage competition (but no losers) 11. Pairing up 12. Recognising physical activity achievement |
| e. Encourage friends and family to engage in physical activity | 1. Audit family and friends’ preferences for physical activity 2. Create protected time for physical activity 3. Make arrangements for physical activity with family and friends 4. Identify opportunities and possibilities 5. Generate awareness and interest 6. Create joint agreements 7. Monitor progress 8. Create a routine 9. Try new things |
